# Visusminderung nach Impfung?

**DOI:** 10.1007/s00347-021-01539-6

**Published:** 2021-12-08

**Authors:** Christiane Kesper, Thomas Kegel, Anja Viestenz, Arne Viestenz

**Affiliations:** 1 Klinik und Poliklinik für Augenheilkunde, Universitätsklinikum Halle (Saale), Martin-Luther-Universität Halle-Wittenberg, Ernst-Grube Str. 40, 06120 Halle (Saale), Deutschland; 2 Klinik für Innere Medizin IV, Universitätsklinikum Halle (Saale), Martin-Luther-Universität Halle-Wittenberg, Halle (Saale), Deutschland

## Anamnese

Eine 38-jährige Patientin stellte sich mit einer seit 3 Stunden bestehenden linksseitigen Visusminderung vor. Eine Amblyopie bestünde nicht. Sie sei kurzsichtig, anderweitige Augenerkrankungen oder Allgemeinerkrankungen bestünden nicht. Lediglich sei eine Hysterektomie aufgrund einer intraepithelialen Neoplasie Grad III der Zervix erfolgt. Sie habe 3 gesunde Kinder, bei und nach deren Geburt es keine Komplikationen gegeben habe. Vor 10 Tagen habe sie ihre erste COVID-19-Impfung mit dem Wirkstoff AZD1222 erhalten.

## Klinischer Befund und Diagnostik

**Figure 1:**
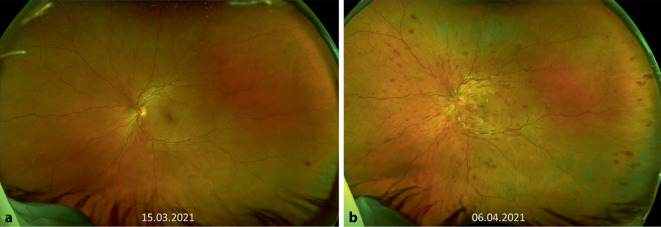

**Figure 2:**
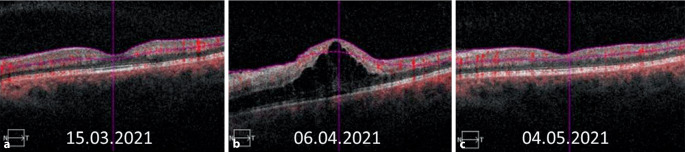

**Figure 3:**
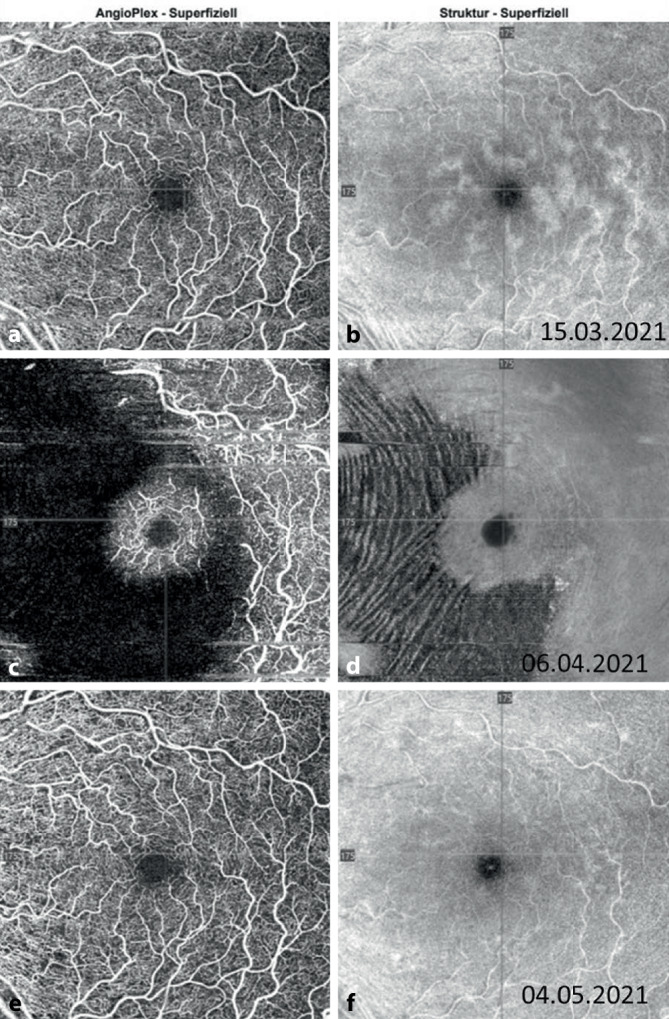

Der bestkorrigierte Visus betrug am rechten Auge 1,0 und am linken Auge 0,05 bei Augeninnendruckwerten von rechts 14 und links 15 mm Hg. Es zeigte sich ein relatives afferentes Pupillendefizit am linken Auge. Die Motilität war frei. Spaltlampenmikroskopisch zeigte sich beidseits der vordere Augenabschnitt regelrecht. Fundoskopisch ließen sich am rechten Auge eine randscharfe und vitale Papille und eine unauffällige Makula bei zirkulärer Netzhautanlage und Tortuositas der Gefäße darstellen. Am linken Auge zeigten sich eine randunscharfe, vital gefärbte Papille mit einer Randblutung, intraretinalen Punktblutungen sowie eine ausgeprägte Tortuositas (Abb. 1a). In der optischen Kohärenztomographie zeigte sich eine intakte foveale Senke (Abb. 2a). Sonographisch bestand kein Anhalt für eine posteriore Skleritis. Die Angio-OCT-Untersuchung zeigte eine noch intakte superfizielle makuläre Perfusion (Abb. 3a, b). Aufgrund des zeitlichen Zusammenhangs mit der Impfung wurden die von der Gesellschaft für Thrombose und Hämostaseforschung (GTH) empfohlenen Untersuchungen (Plättchenfaktor-4-Antikörper-Test und Heparin-induzierter Plättchenaktivierungstest) durchgeführt, welche unauffällig waren. Auch eine Thrombozytopenie bestand nicht, erhöhte D‑Dimere ließen sich nicht nachweisen. Jedoch bestand eine Erhöhung der Anticardiolipin-IgM-Antikörper bei unauffälligen IgG-Antikörpern.

## Wie lautet Ihre Diagnose?

## Therapie und Verlauf

Der schlechte Visus zur Aufnahme erklärte sich am ehesten dadurch, dass eine insgesamt verminderte okuläre Perfusion vorlag. Definitive Zeichen für einen manifesten Zentralvenenverschluss zeigten sich zu diesem Zeitpunkt nicht. Aufgrund des jungen Alters der Patientin sowie einer manifesten ausgeprägten Visusminderung erfolgte nach Rücksprache mit den Hämostaseologen umgehend die PTT-kontrollierte intravenöse Vollheparinisierung mittels unfraktionierten Heparins (UFH) (PTT-Zielbereich 60–80) zur therapeutischen Antikoagulation als Therapieversuch. Ziel war es, die vollständige Thrombosierung zu verhindern. Zur weiteren Neuroprotektion wurde außerdem eine Lokaltherapie mit Brimonidin und Dorzolamid begonnen sowie eine intravenöse Therapie mit Prednisolon eingeleitet. Es erfolgte gemäß den Empfehlungen der GTH eine umfangreiche Ursachendiagnostik, welche eine MRT-Untersuchung des Kopfes, eine CT-Angiographie des Thorax, eine Karotisdopplersonographie, Abdomensonographie, Langzeitblutdruck- und EKG-Untersuchungen beinhaltete. Hier zeigten sich bis auf eine anlagebedingte Hypoplasie des Sinus transversus keine Auffälligkeiten. Weiterhin wurde eine umfassende Gerinnungsdiagnostik eingeleitet. Noch am Aufnahmetag kam es zu einem Visusanstieg auf 1,0 p. Nach 4 Tagen wurde die intravenöse Vollheparinisierung auf eine subkutane Applikation von Enoxaparin 2‑mal täglich (gewichtsadaptiert therapeutisch) für 10 Tage umgestellt. Danach erfolgte die Umstellung auf eine orale Antikoagulation mit Phenprocoumon in Kombination mit ASS nach hämostaseologischer Empfehlung zur Prophylaxe, da initial Anticardiolipin-IgM-Antikörper nachweisbar waren, welche mit einem insgesamt erhöhten Thromboserisiko und einem Antiphospholipidsyndrom einhergehen. Nach Entlassung der Patientin erfolgten regelmäßige Verlaufskontrollen. Es zeigte sich insgesamt eine Befundbesserung.

**Figure 4:**
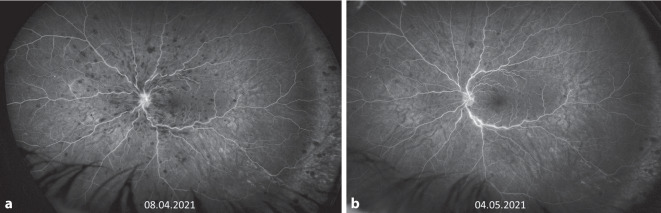

Eine notfallmäßige Vorstellung erfolgte 24 Tage nach Erstereignis mit erneuter Visusminderung auf 0,25 links. Fundoskopisch bot sich nun unter laufender Antikoagulation und Aggregationshemmung das Vollbild eines Zentralvenenverschlusses mit ausgeprägtem zystoidem Makulaödem (Abb. 1b, 2b und 3c, d) am linken Auge. Es erfolgte die erneute Umstellung auf eine PTT-kontrollierte intravenöse Vollheparinisierung mit UFH nach initialer Applikation des Prothrombinkomplexes zur Neutralisierung der begonnenen Marcumar-Therapie nach hämostaseologischer Empfehlung. Diese Umstellung erfolgte, da die Blutungsneigung unter Marcumar deutlich erhöht und unter Heparinisierung besser steuerbar ist. Am Folgetag wurde leitliniengerecht am linken Auge intravitreal Aflibercept appliziert. In der spezifischen Gerinnungsdiagnostik waren inzwischen eine heterozygote (1691G>A) Mutation im Gen für den Gerinnungsfaktor V sowie eine homozygote (677C>T) Mutation des Gens für die Methylen-Tetrahydrofolat-Reduktase (MTHFR) nachgewiesen worden. Heparininduzierte Plättchenantikörper/Anti-Plättchenfaktor-4-Antikörper konnten erneut nicht nachgewiesen werden. Die initial erhöhten Anti-Cardiolipin-IgM-Antikörper waren wieder normwertig, sodass kein Anhalt für ein Antiphospholipidsyndrom bestand. Aufgrund der mittlerweile nachgewiesenen thrombophilen Risikofaktoren wurde im Verlauf eine therapeutische Antikoagulation mit Tinzaparin in Kombination mit ASS empfohlen. Bereits nach der ersten Eingabe von Aflibercept stieg der Visus auf 1,0 an. Das Makulaödem war regredient, die retinalen Blutungen befanden sich in Resorption (Abb. 2c). Auch die peripher nicht perfundierten Areale in der FAG waren wieder perfundiert (Abb. 4a, b). Die Angio-OCT-Untersuchung zeigte ebenfalls eine verbesserte Perfusion (Abb. 3e, f).

## Hintergrund

Eine Zentralvenenthrombose tritt am häufigsten zwischen dem 60 und 70. Lebensjahr auf. Risikofaktoren für die Entstehung einer solchen Thrombose sind unter anderem arterielle Hypertonie, Diabetes mellitus, Nikotinabusus und okuläre Erkrankungen, wie beispielsweise Glaukome oder eine Vaskulitis [1, 2]. Anzeichen für diese Risikofaktoren lagen bei der vorgestellten Patientin nicht vor. Jedoch konnte eine heterozygote Faktor-V-Leiden-Mutation diagnostiziert werden, welche bis dahin nicht bekannt war. Der Zusammenhang zwischen einem Faktor-V-Leiden und einem ZVV wird jedoch kontrovers diskutiert [3].

Ebenfalls neu diagnostiziert wurde eine homozygote MTHFR-Mutation, welche zu einer Hyperhomozysteinämie führen kann. Diese steht jedoch in Korrelation zu der Entstehung eines ZVV, sodass hier ein Risikofaktor vorliegt [4].

Herauszustellen ist, dass es nach dem Auftreten der Präthrombose zu einem raschen Wiederanstieg der Sehleistung nach Vollheparinisierung kam. Bei der Therapie wurde gemäß der Studie von Ageno et al. [5] vorgegangen und Heparin für 14 Tage in therapeutischer Dosierung gegeben. Es handelte sich hierbei um einen erfolgreichen Therapieversuch, welcher mit der Zustimmung der Patientin unternommen wurde. Studien konnten hier jedoch noch keine ausreichende Wirksamkeit belegen. Die initiale Nutzung eines Heparinperfusors erfolgte zur besseren Kontrollierbarkeit der Ziel-PTT.

**Diagnose:** Prästase

Die Rolle der COVID-19-Impfung gilt es in diesem Fall zu diskutieren. Die Patientin zeigte vor der Präthrombose bzw. der Zentralvenenthrombose keinerlei thrombotische Ereignisse. Auch Fehlgeburten oder Beinvenenthrombosen, welche mit einer Faktor-V-Leiden-Mutation vergesellschaftet sind, lagen nicht vor [6]. Der COVID-19-Impfstoff AZD1222, welchen die Patientin 10 Tage vor Erstereignis erhalten hatte, steht im Verdacht, Thrombosen zu verursachen. Die Greifswalder Arbeitsgruppe um Greinbacher fand diesbezüglich heraus, dass die Impfung vermutlich eine inflammatorische Reaktion und Immunstimulation auslöst, welche dann zu einer Antikörperbildung gegen Plättchenantigene führt. Diese Antikörper vermitteln anschließend eine Kettenreaktion, wie sie von der Heparin-induzierten Thrombozytopenie bekannt ist [7]. Die im Rahmen der Studie untersuchten Patienten wiesen 4 bis 16 Tage nach der Impfung mit AZD1222 ein thrombotisches Ereignis, zumeist eine Sinusvenenthrombose auf. Die diesbezüglich von der GTH empfohlenen Untersuchungen zur Abklärung (Plättchenfaktor-4-Antikörper-Test und Heparin-induzierter Plättchenaktivierungstest) wurden mit dem Blut der Patientin durchgeführt, waren jedoch unauffällig. Auch eine Thrombozytopenie bestand nicht. Es bleibt abzuwarten, ob noch weitere Mechanismen zur Thromboseentstehung nach AZD1222-Impfung entdeckt werden, welche evtl. auch auf die vorgestellte Patientin zutreffen. Eine Möglichkeit wäre beispielsweise eine Triggerung durch die postvakzinale Immunreaktion des Thrombophilierisikos durch die MTHFR-Mutation und das Faktor-V-Leiden. Dies bleibt aber nachzuweisen.

## Fazit für die Praxis

Eine Präthrombose oder eine Zentralvenenthrombose könnte eine mögliche Komplikation einer COVID-19-Impfung darstellen. Hier gilt es, bei entsprechender Patientenklientel den Impfstatus zu erfragen, aber auch die weiterführende Umfelddiagnostik wie die in diesem Fall ebenfalls wegweisende Gerinnungsdiagnostik nicht außer Acht zu lassen. Hervorzuheben ist weiterhin die in diesem Fall visusverbessernde Vollheparinisierung nach dem Initialereignis, die bei geeigneten Patienten in Erwägung gezogen werden kann.
